# Statewide implementation & sustainment of evidence-based treatment using learning collaboratives: A five-year mixed-methods study

**DOI:** 10.1186/1748-5908-10-S1-A78

**Published:** 2015-08-20

**Authors:** Jason Lang, Christopher Bory

**Affiliations:** 1 Child Health and Development Institute, Farmington, CT, 06032, USA; 2 Department of Psychiatry, UConn Health Center, Farmington, CT 060, USA; 3 Child Study Center, Yale University, New Haven, CT 06519, USA

## 

Connecticut was among the first states to begin using learning collaboratives (LCs) for the statewide implementation of a behavioral health EBP in 2007. Based upon the Institute for Healthcare Improvement's Breakthrough Series Collaborative, LCs are an intensive quality improvement approach now being used to disseminate EBPs nationally. However, there has been little research on the LC model for EBP dissemination. This presentation summarizes mixed-methods data from a statewide dissemination of Trauma Focused Cognitive Behavioral Therapy (TF-CBT) to 16 community-based clinics using LCs. Pre-and post-implementation measures of staff attitudes towards EBPs and perceptions of organizational support were collected. Measures of acceptability of the LC model and satisfaction were collected post-implementation, and focus groups were held for clinicians, supervisors, and administrators. Monthly data on fidelity, penetration, and child level outcomes have been collected for 5 years post-implementation. Implementation outcome data from over 300 staff trained at the 16 agencies, who have served 2,300 children and families, will be presented. Results suggest that the LC is an effective, yet time intensive, implementation approach that resulted in sustained use of an EBP for several years despite significant challenges. Staff reported variability in the utility of various components of the model, but overall reported it to be an effective implementation approach. Staff reported significant improvements in attitudes about EBPs from pre-to post-implementation. Implementation costs, staff turnover, and sustainability were identified as significant challenges. Child outcome data indicate significant clinical improvements in PTSD and depression symptoms. This research provides support for the use of LCs as a dissemination model. The results suggest potential strategies for evaluating modifications to LCs to improve dissemination, and how to evaluate specific implementation strategies from LCs in other dissemination strategies. This study was funded through a contract with the Connecticut Department of Children and Families.

